# Dysregulation of mitochondrial dynamics mediated aortic perivascular adipose tissue-associated vascular reactivity impairment under excessive fructose intake

**DOI:** 10.1186/s12986-023-00776-7

**Published:** 2024-01-02

**Authors:** Kay L. H. Wu, Chih-Wei Wu, Lee-Wei Chen, Hsiao-Huang Chang, Ching-Li Cheng, Cai-Yi Wu, Yu-Chi Lee, I-Chun Chen, Chun-Ying Hung, Wen-Chung Liu

**Affiliations:** 1 Institute for Translational Research in Biomedicine, Kaohsiung Chang Gung Memorial Hospital, Kaohsiung, Taiwan, ROC; 2 Department of Senior Citizen Services, National Tainan Institute of Nursing, Tainan, Taiwan, ROC; 3 Plastic Surgery, Kaohsiung Veterans General Hospital, Kaohsiung, Taiwan, ROC; 4 Department of Counseling, National ChiaYi University, Chiayi, Taiwan, ROC; 5 Department of Surgery, Kaohsiung Veterans General Hospital, Kaohsiung, Taiwan, ROC; 6 Institute of Emergency and Critical Care Medicine, National Yang-Ming Chiao Tung University, Taipei, Taiwan, ROC; 7 Department of Biological Sciences, National Sun Yat-Sen University, Kaohsiung, Taiwan, ROC; 8 Department of Surgery, School of Medicine, Taipei Medical University, Taipei, Taiwan, ROC; 9 Division of Cardiovascular Surgery, Department of Surgery, Taipei Veterans General Hospital, Taipei, Taiwan, ROC; 10 Department of Nursing, National Tainan Institute of Nursing, Tainan, Taiwan, ROC; 11 Department of Surgery, School of Medicine, National Yang-Ming University, Taipei, Taiwan, ROC; 12 Institute of Biomedical Sciences, National Sun Yat-Sen University, Kaohsiung, Taiwan, ROC; 13 School of Medicine, College of Medicine, National Sun Yat-Sen University, Kaohsiung, Taiwan, ROC

**Keywords:** High fructose diet, Perivascular adipose tissue, Adipose whitening, Mitochondrial dynamics, Vascular reactivity

## Abstract

Excessive fructose intake presents the major risk factor for metabolic cardiovascular disease. Perivascular adipose tissue (PVAT) is a metabolic tissue and possesses a paracrine function in regulating aortic reactivity. However, whether and how PVAT alters vascular function under fructose overconsumption remains largely unknown. In this study, male Sprague-Dawley rats (8 weeks old) were fed a 60% high fructose diet (HFD) for 12 weeks. Fasting blood sugar, insulin, and triglycerides were significantly increased by HFD intake. Plasma adiponectin was significantly enhanced in the HFD group. The expression of uncoupling protein 1 (UCP1) and mitochondrial mass were reduced in the aortic PVAT of the HFD group. Concurrently, the expression of peroxisome proliferator-activated receptor-γ coactivator 1α (PGC-1α) and mitochondrial transcription factor A (TFAM) were suppressed. Furthermore, decreased fusion proteins (OPA1, MFN1, and MFN2) were accompanied by increased fission proteins (FIS1 and phospho-DRP1). Notably, the upregulated α-smooth muscle actin (α-SMA) and osteocalcin in the PVAT were concurrent with the impaired reactivity of aortic contraction and relaxation. Coenzyme Q_10_ (Q, 10 mg/100 mL, 4 weeks) effectively reversed the aforementioned events induced by HFD. Together, these results suggested that the dysregulation of mitochondrial dynamics mediated HFD-triggered PVAT whitening to impair aortic reactivity. Fortunately, coenzyme Q_10_ treatment reversed HFD-induced PVAT whitening and aortic reactivity.

## Background

Patients with metabolic syndrome (MetS) appear to develop vascular complications [1]. According to previous studies in both human and animal models, excessive fructose ingestion induces MetS, including central obesity [2], hypertriglyceridemia [3], insulin resistance [4], and hypertension [5–8]. However, the adverse effect of the perivascular microenvironment on vascular dysfunction remains elusive.

The perivascular adipose tissue (PVAT) of the thoracic aorta more closely resembles brown adipose tissue (BAT) [9, 10], which contains multilocular adipocytes with ample mitochondria and uncoupling protein 1 (UCP 1) [10, 11]. In contrast, white adipose tissue (WAT) has larger unilocular adipocytes [12], low expression of UCP 1, and fewer mitochondria [13, 14]. Converting BAT to a WAT-like appearance with decreased UCP1 and reduced mitochondrial mass is defined as BAT whitening. Fructose is a lipogenic carbohydrate used as a common sweetener in beverages and desserts. Although adipose tissue whitening has been linked to the progression of metabolic disorders, whether a high-fructose diet (HFD) turns PVAT into whitening and the underlying mechanisms are inconclusive.

Mitochondrial mass is strictly regulated by fission, fusion, biogenesis, and mitophagy to maintain the homeostasis of bioenergetics. Mitochondrial biogenesis is a process to generate new mitochondria. The reduction of mitochondrial biogenesis would lead to reduced mitochondrial mass. As a master regulator of mitochondrial biogenesis, peroxisome proliferator-activated receptor-γ coactivator -1α (PGC-1α) encodes mitochondrial transcription factor A (TFAM), which is the final effector of mitochondrial DNA (mtDNA) transcription and replication [15–17]. In the liver of rats fed a fructose-rich diet, the levels of PGC-1α and mtDNA were reduced [18]. It is possible that the HFD might reduce the mitochondrial mass of PVAT by downregulating these regulators of mitochondrial biogenesis.

To mitigate stress and maintain the mitochondrial capacity of genetic and biochemical homogeneity, fusion dilutes the increased reactive oxygen species and mutated mtDNA by mixing the contents of partially impaired mitochondria as a form of complementation. The mitochondrial fusion process includes the fusion of the outer membranes by mitofusin 1 (MFN1) and MFN2 [19] and of the inner membranes by optic atrophy 1 (OPA1) [20]. Recently, the suppression of mitochondrial fusion proteins has been linked to adipose whitening [21]. This evidence might link the dysregulation of mitochondrial fusion to HFD-associated PVAT whitening.

In addition, fission, known as mitochondrial fragmentation, enables mitochondrial clearance of dysfunctional mitochondria [22]. Dynamin-related protein 1 (DRP1) binds to the mitochondrial fission protein (FIS1) at the periphery of mitochondria and facilitates the division of damaged mitochondria to a smaller fraction for mitophagy. Cooperating with fusion, mitochondrial fission plays critical roles in maintaining the function of mitochondria under metabolic stresses [23]. An imbalance of fusion and fission could lead to a dysregulation of mitochondrial quality.

PVAT surrounds the arteries and directly contacts the vascular wall. Instead of structural support, PVAT is considered an endocrine organ that releases bioactive molecules that directly modulate vascular tone in a paracrine fashion to increase [24, 25] or attenuate [26] vasoconstriction. It is conceivable that PVAT whitening induced by HFD might contribute to the progression of metabolic vascular dysfunction. Nonetheless, the mechanism remains largely unclear.

Herein, we fed Sprague-Dawley rats HFD to investigate the whitening of thoracic aortic PVAT, the role of mitochondria in the progression of PVAT whitening, and the contribution of whitened PVAT to changes in aortic reactivity.

## Materials and methods

### Animals

**Fig. 1:**
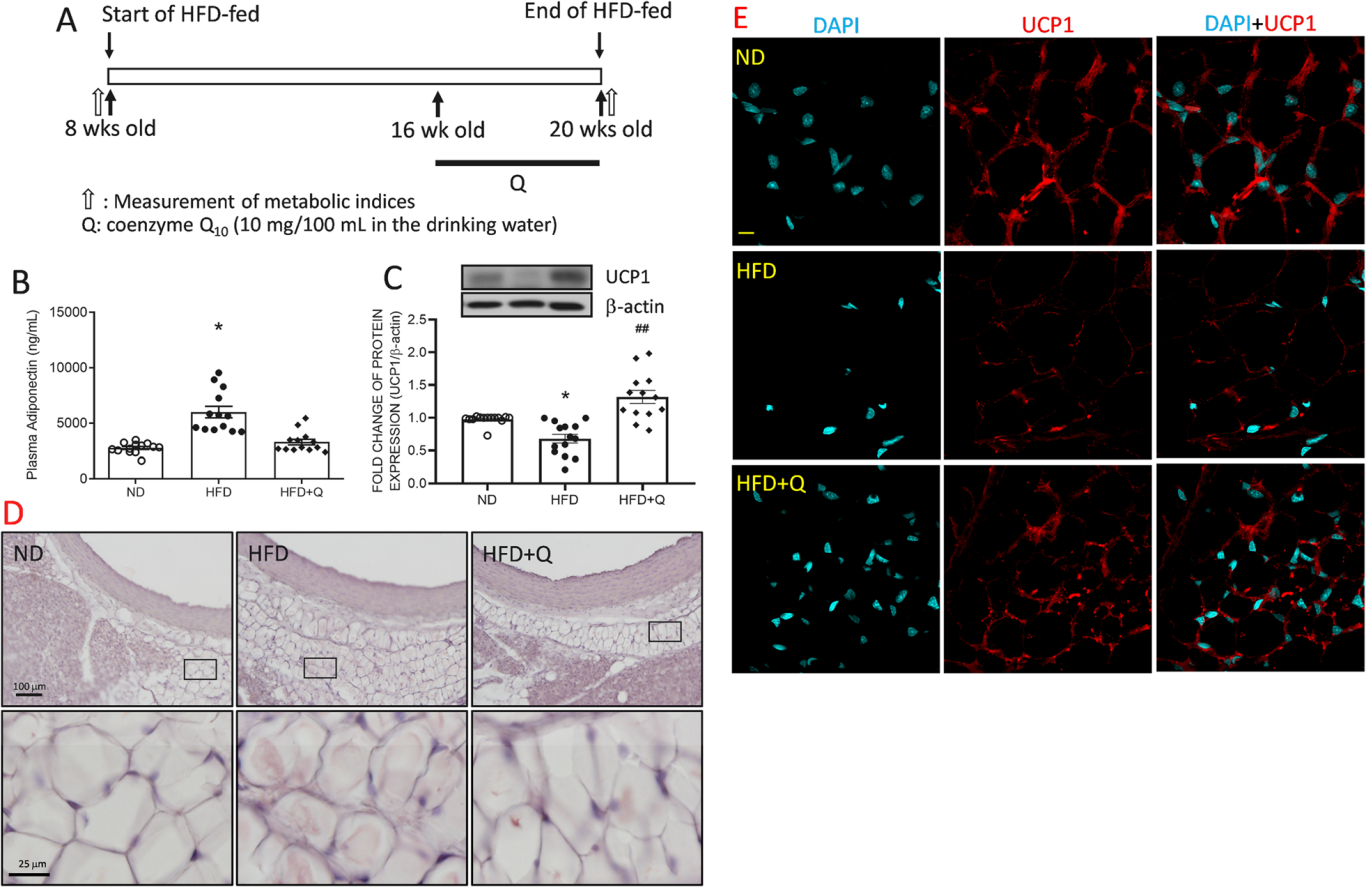
Coenzyme Q_10_ treatment reversed the HFD-induced suppression of adipose browning factors in the aortic PVAT. **A** Schematic illustration of the study design to evaluate high fructose diet (HFD)-induced metabolic changes. The special diet was started at 8 weeks old for 3 months. The metabolic indices were measured before and at the end of the special diet at 8 and 20 weeks of age. Coenzyme Q_10_ was applied to drinking water (10 mg/100 mL) for 4 weeks from 16 to 20 weeks of age. Then, **B** plasma adiponectin, **C** representative gel and densitometric analyses of intracellular UCP1, (D) the representative images of Hematoxylin & Eosin (H&E) staining, and **E** representative images of UCP1 (red) in aortic PVAT at the age of 20 weeks. Values are the mean ± SEM, n = 14 for each group. *P < 0.05 versus the ND group and ^***##***^*P* < 0.01 versus the HFD group in the post hoc Tukey’s multiple range test. ND: regular diet, HFD, high fructose diet, HFD + Q, HFD with coenzyme Q_10_. DAPI, 4′,6-diamidino-2-phenylindole, a blue-fluorescent nuclear stain. PVAT, aortic perivascular adipose tissue, UCP, uncoupling protein, β-actin as the internal control of Western blot analyses. Scale bars: 100 μm (Fig. 1D, upper panel), 50 μm (**D**, lower panel), 10 μm (**E**)

Male, adult Sprague-Dawley rats (SD rats, 6 weeks old) were purchased from the Experimental Animal Center, National Science Council, Taiwan. Animals were allowed to acclimatize in a temperature (22 ± 1 °C), humidity (55 ± 5%) and light (12:12 light–dark cycle, lights on from 0:800) controlled room in a certified animal facility for at least 14 days before the experiments. All experiments were carried out in accordance with the guidelines for animal experimentation endorsed by our institutional animal care and use committee (IACUC; 2019022602, 2019060701). These male rats were randomly assigned to feed with regular chow (ND) or a high fructose diet (HFD). For the HFD group, animals received 60% fructose (Envigo, Indianapolis, UK; Table 1) as the sole food source from 2 months old for 3 months. The animals in the ND group received regular chow (Purina, MO, USA; Table 1). Both food and water were provided ad libitum. Waistline, as well as the volumes of food and water intake, were measured and recorded twice a week. Coenzyme Q_10_ (Ubidecarenone, 10 mg/100 mL) [27] was applied to drinking water for 4 weeks from 16 to 20 weeks of age following the medicine instruction. The metabolic indices of each animal were measured before the experiment was processed. Only those with similar levels of metabolic indices were used for the study. The illustrated scheme of the study design is shown in Fig. 1A.

**Table 1:** The dietary contents of the regular diet (ND) and high fructose diet (HFD)

	Regular chew (ND)	High fructose diet (HFD, 60%)
Fructose	–	600.0 g/kg
Lard	50.0 g/kg	50.0 g/kg
Casein	232.3 g/kg	207.0 g/kg
Cellulose	51.0 g/kg	79.81 g/kg
DL-Methionine	6.7 g/kg	3.0 g/kg
Mineral Mix Rogers-Harper (170,760)	> 7 g/kg	50.0 g/kg
Zinc carbonate		0.04 g/kg
Vitamin mix Teklad (40,060)		10.0 g/kg
Food color	–	0.15 g/kg (Green)
kcal from		
Carbohydrate	57.996%	66.8%
Fat	13.496%	13.0%
Protein	28.507%	20.2%
Kcal/g	3.35	3.6

### Detection of fasting blood glucose

The level of fasting blood glucose (FBG) was monitored in 8-week-old animals to ensure the same basal level of metabolic indices in both the normal diet (ND) and high fructose diet (HFD) groups before special diet feeding. After 3 months of ND or HFD feeding, FBG was monitored to evaluate the effects of diet. Animals were fasted for at least 15 h for the tests. Blood samples were collected from a punch at the tip of the tail, and glucose levels were analyzed using the glucose oxidase method (Roche, Basel, Switzerland) [28]. Detection was performed before and after 12 weeks of HFD ingestion.

### Insulin enzyme-linked immunosorbent assay

Blood samples collected from the tail artery were centrifuged at 3068 rpm for 10 min to separate the plasma for insulin detection. Fasting plasma insulin (Mercodia, Uppsala, Sweden) was analyzed by means of ELISAs based on a direct sandwich enzyme immunoassay, and two monoclonal antibodies were applied against insulin molecules. During incubation, sample insulin was reacted with peroxidase-conjugated anti-insulin antibodies. The bound conjugate was detected by reaction with 3,3′,5,5′-tetramethylbenzidine. The reaction was stopped by adding stop solution and read by a spectrophotometer at 450 nm. Each assay was performed in triplicate.

### Measurement of fasting plasma triglyceride concentration

To determine the concentration of fasting plasma triglycerides (TG) by a triglyceride assay kit (Randox, Antrim, UK), 10 μl of plasma from the tail artery after 15 h of fasting was incubated with the reaction mixture at 25 °C for 20 min. TG concentrations were detected at 570 nm. Each assay was performed in triplicate.

### Hematoxylin & eosin (H&E) staining

The PVAT samples were flushed with PBS and heparin (250 U/kg) and fixed in 4% paraformaldehyde for 72 h at 4 °C after perfusion. After cryoprotected with 30% sucrose solution, the tissue was embedded in an optimal cutting temperature compound (OCT compound) and sliced with a freezing microtome at 20 μm. The sections were stained with hematoxylin and eosin stain following the user guide (Sigma-Aldrich, USA). The images were observed under a light microscope (BX53, Olympus, Japan).

### Immunofluorescence analyses

PVAT was flushed with PBS and heparin (250 U/kg) and fixed in 4% paraformaldehyde for 72 h at 4 °C after perfusion. Next, the samples were cryoprotected with 30% sucrose solution. The samples were embedded in an optimal cutting temperature compound (OCT compound) and sliced with a freezing microtome at 20 μm. The slices were collected for further staining. For immunofluorescence staining, the slices from each group were labeled alone with UCP1 (1:500, Proteintech, IL, USA). Proper fluorescent-conjugated secondary antibodies (Invitrogen, MA, USA) were applied. To visualize mitochondrial mass, sections from each group were collected and placed onto poly-lysine–coated slides. MitoTracker was applied to the slides for a 2-h incubation at 4 °C. DAPI (4′,6-diamidino-2-phenylindole) was applied as a blue-fluorescent nuclear stain. The images were observed and analyzed by using ImageJ software (NIH, Bethesda, MD).

### Total protein isolation

For Western blotting analyses, tissue samples from PVAT were homogenized with a Dounce grinder with a tight pestle in ice-cold lysis buffer (15 mM HEPES, pH 7.2, 60 mM KCl, 10 mM NaCl, 15 mM MgCl_2,_ 250 mM sucrose, 1 mM EGTA, 5 mM EDTA, 1 mM PMSF, 2 mM NaF, 4 mM Na_3_VO_4_). A mixture of leupeptin (8 μg/mL), aprotinin (10 μg/mL), phenylmethylsulfonyl fluoride (20 μg/mL) and trypsin inhibitor (10 μg/mL) were included in the isolation buffer to prevent protein degradation. The homogenate was centrifuged at 13,500 rpm for 30 min, and the supernatant was collected for protein analysis. The concentration of the total protein extracted was estimated by the Bradford method with a protein assay kit (Bio-Rad, Hercules, CA).

### Western blot analysis

Proteins of interest in PVAT were separated by using 10–12% SDS‒PAGE. Samples from each group contained equivalent total protein concentrations. The electrophoretic proteins were transferred onto polyvinylidene difluoride (PVDF) membranes (Immobilon-P membrane; Millipore; Bedford, MA, USA). Membranes were probed with specific antibodies against Uncoupling Protein 1 (UCP1, 1:1000, Proteintech, IL, USA), mitochondrial respiratory complex 1 (mt CPX1, 1:1000, Thermo Fisher Scientific, MA, USA), mt CPX2 (1:1000, Thermo Fisher Scientific), mt CPX3 (1:1000, Thermo Fisher Scientific), mt CPX4-1 (1:1000, Thermo Fisher Scientific), mt CPX4-2 (1:1000, Thermo Fisher Scientific), mt CPX5 (1:1000, Thermo Fisher Scientific), peroxisome proliferator-activated receptor γ coactivator 1-α (PGC-1α, 1:1000, Santa Cruz Biotechnology, Inc.), mitochondrial transcription factor A (TFAM, 1:1000, BioVision Inc., CA, USA), optic atrophy 1 (OPA1, 1:1000, Abcam), mitofusin 1 (MFN1, 1:1000, Abcam), MFN2 (1:1000, Cell Signaling Technology, MA, USA), mitochondrial fission 1 protein (FIS1, 1:1000, Merck KGaA, Darmstadt, Germany), dynamin-related protein 1 (DRP1, 1:1000, Cell Signaling Technology), phospho(p)-DRP1 (p-DRP1, 1:1000, Cell Signaling Technology), α-SMA (1:1000, Abcam, Cambridge, UK), and osterocalcin (1:1000, Abcam)[29]. The membranes were then incubated with the appropriate horseradish peroxidase–conjugated secondary antibody. Specific antibody-antigen complexes were detected using an enhanced chemiluminescence Western blot detection system (Thermo Fisher Scientific). The amounts of detected proteins were quantified by ImageJ software (NIH, MD, USA) and were expressed as the ratio to β-actin protein.

### Intracellular ATP content measurement

The ATP content of PVAT was determined by an ATP colorimetric assay kit (Biovision). Total protein samples from PVAT were centrifuged at 13,500 rpm, 15 min. 10 μl supernatant was incubated with ATP reaction mixture for 30 min. The ATP levels were detected at 570 nm using a microplate reader (Thermo Fisher Scientific Inc.). ATP levels were normalized to the protein concentration of the samples. Protein concentrations were determined by the Bradford analysis. All experiments were repeated in triplicates.

### Genomic DNA extraction

A QIAamp DNA Mini Kit (QIAGEN, Hilden, Germany) was used to isolate PVAT genomic DNA according to the manufacturer's protocol. In brief, PVAT tissues were incubated at 56 °C overnight with 180 μL of buffer ATL and proteinase K. RNase A was then added for 2 min at room temperature. Next, 200 μL of Buffer AL was added to each sample and incubated at 70 °C for 10 min. Then, 200 μL of 100% ethanol was added to the sample and gently mixed. Then, the mixture was applied to the QIAamp Mini spin column for centrifugation at 6000×*g* for 1 min. The column was then washed by adding 500 μL Buffer AW1 and centrifuged at 6000×*g* for 1 min. Next, 500 μL Buffer AW2 was applied for centrifugation at 20,000×*g* for 3 min, followed by another centrifugation at 20,000×*g* for 1 min. The column was incubated with 20–30 μL water at 65 °C for 5 min and then centrifuged at 6000×*g* for 1 min to obtain the genomic DNA for concentration detection [30].

### Quantitative analysis of mitochondrial DNA copies relative to nuclear DNA

The ratio of cDNA amplified from mitochondrial DNA (mtDNA)-encoded NADH dehydrogenase subunit 1 (ND1) to nucleus-encoded 18S rRNA genes were evaluated as described in a previous study [30]. In brief, primers for the ND1 probe corresponded to nucleotides 389–408 (forward) and 572–592 (reverse; PCR product of 200 base pairs) of the rat mitochondrial genome (Chromosome MT—NC_001665.2). Primers for the 18S probe corresponded to nucleotides 681–702 (forward) and 864–884 (reverse; PCR product of 200 base pairs) of the rat nuclear genome (Chromosome 14—NC_005113.3). The primer sequences were as follows: ND1, Forward (5′–3′) TCGGAGCCCTACGAGCCGTT/Reverse (5′–3′) AGGGAGCTCGATTTGTTTCTG; 18S rRNA, Forward (5′–3′) TAGTTGGATCTTGGGAGCGGG/Reverse (5′–3′) CCGCGGTCCTATTCCATTATT. 18S rRNA served as a control. Quantitative real-time polymerase chain reaction (PCR) was performed in a Roche LightCycler 480 (Roche Applied Science, Mannheim, Germany) apparatus with the LightCycler 480 SYBR Green I Master kit (Roche Applied Science). Ten nanograms of extracted DNA was mixed with 10 μL LightCycler 480 SYBR Green I Master Mix that contained 5 μmol (final concentration 0.4 μM) of forward and reverse primer in a final volume of 20 μL. The qPCR procedure was as follows: initiation at 50 °C for 2 min; 95 °C for 1 min; 40 cycles of denaturation at 95 °C for 15 s; annealing at 60 °C for 20 s; extension at 72 °C for 15 s; and finally holding at 4 °C. The value was determined for each individual quantitative PCR run. The ΔCt = [Ct_ND1_–Ct_18S_] represents the relative abundance. The quantitative results were expressed as the copy number of mtDNA for each sample by the 2^−ΔCt^ method. Each measurement was at least triplicated and normalized in each experiment against serial dilutions of a control DNA sample.

### Measurement of vascular reactivity

After the animal was anesthetized with pentobarbital (150 mg/kg, peritoneal injection), the thoracic descending aorta was harvested and placed in the oxygenated (95% O_2_ + 5% CO_2_) Krebs' solution. Aortic rings 2 mm long (PVAT removed) were isolated and mounted in organ chambers containing 10 mL Krebs physiological salt solution (PSS: 120 mM NaCl, 5.9 mM KCl, 25 mM NaHCO_3_, 1.2 mM NaH_2_PO_4_, 11.5 mM dextrose, 1.2 mM MgCl_2_ and 2.5 mM CaCl_2_). The chambers were maintained at 37 °C and aerated continuously with 94% O_2_–6% CO_2_. Changes in isometric force were recorded continuously using an isometric force‒displacement transducer (Grass FT03; Grass Instrument, West Warwick, RI). Each ring was gradually stretched to 1.2 g, which allowed for maximal force production. Then, aortic rings were stimulated twice with KCl-PSS (equimolar replacement of NaCl with KCl) to generate reproducible contraction. After washing and a 45-min equilibration period, a dose–response of contraction was generated with cumulative concentrations of the α1-adrenoceptor agonist phenylephrine (PE; 10^9^ to 10^5^ M; Sigma‒Aldrich, St. Louis, MO) to record the vascular contraction. After washing three times, the concentration–response curves of aortic rings were obtained by cumulative addition of acetylcholine (10^9^ to 10^5^ M; Sigma‒Aldrich) to examine aortic relaxation. Papaverine (3 × 10^4^ M; Sigma‒Aldrich) was used to induce complete relaxation of the vessels. All experiments were performed in vessels with intact endothelium [31].

### Statistical analysis

Data are expressed as the means ± SEM. The statistical software GraphPad Prism (La Jolla, CA, USA) was used for data analysis. Student’s unpaired *t* test was used in metabolic indices that involved two groups of animals. For biochemical experiments that involved multiple groups, one-way of variance with repeated measures was used to assess group means followed by Tukey’s multiple range test for post hoc assessment of individual means. *P* < 0.05 was considered statistically significant.

## Results

### Coenzyme Q_***10***_ treatment reversed the high fructose diet suppression of uncoupling protein 1 in aortic perivascular adipose tissue

To ensure that the metabolic status was comparable between groups, fasting blood sugar (FBS), fasting blood insulin (FBI), fasting blood triglyceride (FBTG) and waist circumference were detected at 8 weeks of age before a high-fructose diet (HFD) was fed (study design shown in Fig. 1A). These metabolic indices were detected after 3 months of HFD ingestion (Table 2). The results indicated that FBS, FBI, and FBTG were significantly increased in the HFD group compared with the age-matched ND groups. On the other hand, the waist circumference and body weights were not significantly different between the groups (Table 2). Food intake showed no significant difference between the groups, while water consumption was significantly higher in the HFD group than in the ND group (Table 3). These data indicated that HFD intake for 3 months could induce metabolic syndrome in adult male SD rats before central obesity was induced. Coenzyme Q_10_ (Q) oral application showed no effect on reversing these systemic metabolic indices induced by HFD. On the other hand, the plasma level of adiponectin was significantly increased in the HFD group and was reversed by coenzyme Q_10_ (Fig. 1B).

**Table 2:** The metabolic indices of rats at 8 and 20 months of age after a 3-month regular diet (ND) and high fructose diet (HFD)

Age (weeks old)	group	Fasting blood sugar (mmol/L)	Fasting blood triglyceride (mmol/L)	Fasting blood insulin (μU/mL)	Waistline (cm)	Body weight (g)
8	ND	4.36 ± 0.163	1.58 ± 0.044	3.49 ± 0.383	12.53 ± 0.117	309.4 ± 6.437
	HFD	4.42 ± 0.146	1.65 ± 0.060	2.89 ± 0.235	12.45 ± 0.351	332.0 ± 18.22
20	ND	4.60 ± 0.153	1.79 ± 0.072	7.89 ± 0.450	15.32 ± 0.209	508.0 ± 22.61
	HFD	5.47 ± 0.186*	2.15 ± 0.048*	13.26 ± 0.757***	15.22 ± 0.391	476.5 ± 17.10
	HFD + Q	5.44 ± 0.170*	2.26 ± 0.068^##^	14.53 ± 0.845^###^	15.93 ± 0.498	518.7 ± 36.79

Values are the mean ± SEM, n = 14 for each group

ND, regular diet, HFD, high fructose diet, Q, coenzyme Q_10_

*P < 0.05, **P < 0.01, and ***P < 0.001 versus the ND group, ^##^*P* < 0.01, and ^###^*P* < 0.001 versus the HFD group in the *Student’s t* test (8 weeks old) or in the post hoc Sidak’s multiple range test. (20 weeks old)

**Table 3:** The food intake and drinking water volumes of rats from 8 to 20 months old fed a regular diet (ND) and a high fructose diet (HFD)

group	Food intake (g/day/rat)	Water consuming volume (mL/day/rat)
ND	13.46 ± 0.8065	23.28 ± 1.440
HFD	13.90 ± 0.1751	31.78 ± 1.363**
HFD + Q	14.63 ± 0.2109	25.64 ± 2.036^**#**^

Values are the mean ± SEM, n = 14 for each group. and **P < 0.01 versus the ND group

ND, regular diet, HFD, high fructose diet, Q, coenzyme Q_10_

^**#**^*P* < 0.05 versus the HFD group in the post hoc Sidak’s multiple range test

Brown adipose tissue (BAT) whitening has been identified in the type 2 diabetes model [32]. BAT is characterized by smaller lipid droplets and a higher number of mitochondria as well as changes in lipid droplet size. According to the H&E staining images of aortic perivascular adipose tissue (PVAT), a remnant of central lipid droplet was observed in the HFD group when compared with the ND or HFD + Q groups (Fig. 1D). The changes in lipid droplet size suggest BAT transformation. To examine whether aortic PVAT was whitened by HFD, the level of UCP1 [33] was detected by Western blotting and immunofluorescence. The results indicated that UCP1 expression (Fig. 1C) in aortic PVAT was downregulated. Moreover, the intensity of UCP1 fluorescence (Fig. 1E, red) was reduced in the HFD group compared with the ND group. Fortunately, coenzyme Q_10_ treatment effectively reversed the suppression of UCP1 to the ND level (Fig. 1). UCP1 is primarily located in the mitochondria. These results further implied that mitochondrial mass might be reduced in aortic PVAT by HFD intake.

### Coenzyme Q_***10***_ treatment reversed the mitochondrial mass and the expression of respiratory proteins in aortic perivascular adipose tissue triggered by high fructose diet ingestion

**Fig. 2:**
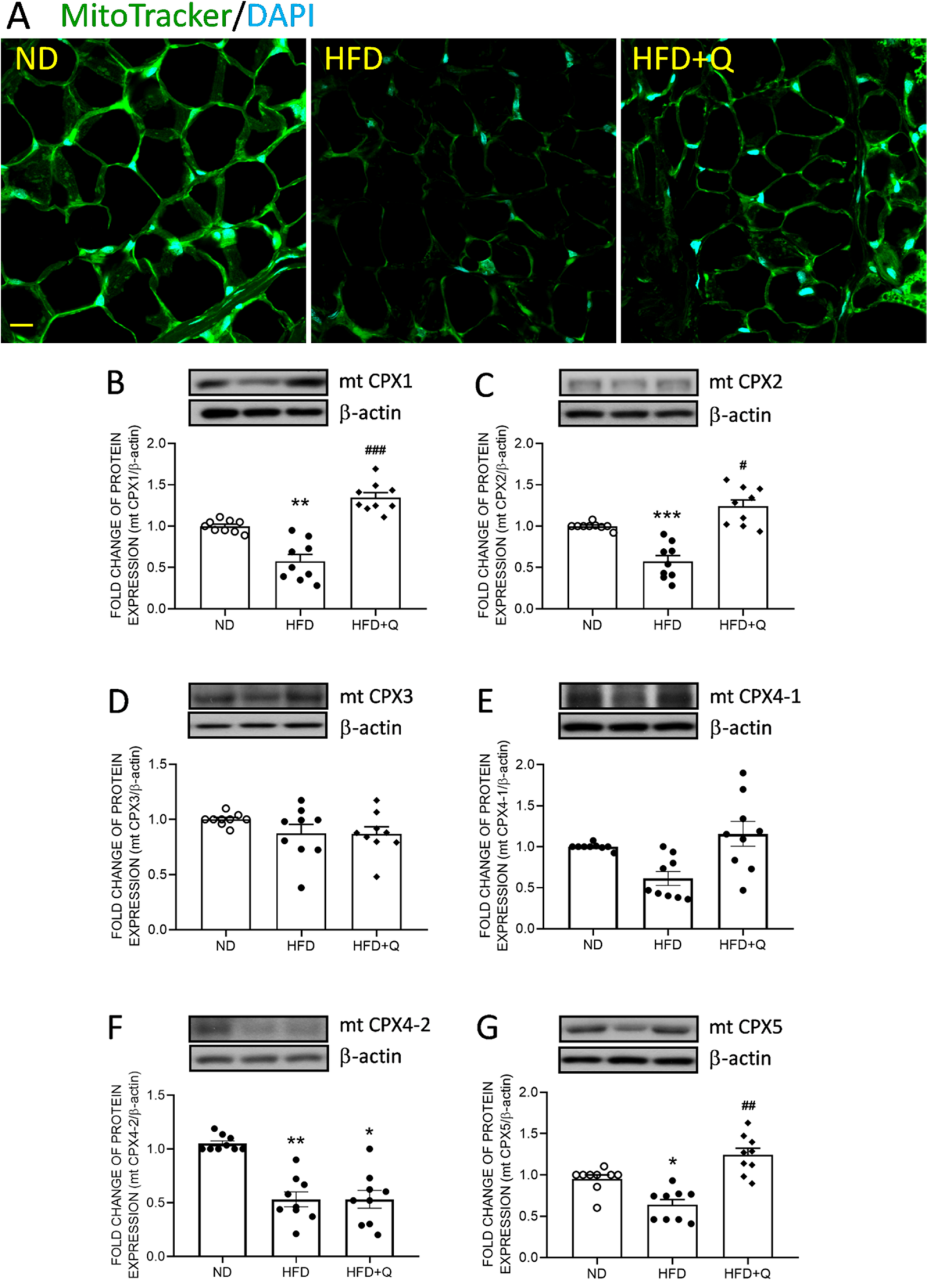
Coenzyme Q_10_ reversed the HFD-reduced mitochondrial mass and respiratory complexes in aortic PVAT. **A** Mitochondrial mass (green, stained by MitoTracker) and representative gel and densitometric analyses of **B** mt CPX1, **C** mt CPX2, **D** mt CPX3, **E** mt CPX4-1, **F** mt CPX4-2, and **G** mt CPX5 in the aortic PVAT of the ND, HFD and HFD + Q groups at the age of 20 weeks. Values are the mean ± SEM, n = 14 for each group. *P < 0.05, **P < 0.01, ***P < 0.001 versus the ND group and ^***#***^*P* < 0.05, ^##^*P* < 0.01, ^###^*P* < 0.001 versus the HFD group in the post hoc Tukey’s multiple range test. ND, normal diet, HFD, high fructose diet, HFD + Q, HFD with coenzyme Q_10_. mt CPX, mitochondrial respiratory complex subunit, β-actin as the internal control of Western blot analyses. Scale bars: 10 μm

Whitened adipocytes have low mitochondrial mass [13, 14]. The immunofluorescence images revealed that the fluorescence intensity of MitoTracker (an index of mitochondrial mass; Fig. 2A, green) was reduced in aortic PVAT from the HFD group compared with the ND group. These results of UCP1 and mitochondrial mass suggested that the PVAT was whitened by HFD intake in 3 months. In addition, the results from Western blot analyses indicated that the expression of mitochondrial respiratory complex 1 (mt CPX1, Fig. 2B), mt CPX2 (Fig. 2C), mt CPX4-2 (Fig. 2F), and mt CPX5 (Fig. 2G) in aortic PVAT was suppressed, while mt CPX4-1 (Fig. 2E) showed a decreasing trend in the HFD group. These results further suggested that HFD consumption reduced mitochondrial mass concurrent with a decrease in mitochondrial respiratory complexes in aortic PVAT. Fortunately, coenzyme Q_10_ treatment effectively reversed the suppression of mt CPX1, 2 and 5 to the ND level (Fig. 2). These data further implied dampened mitochondrial biogenesis under HFD intake. On the other hand, no significant difference was detected in mt CPX3 (Fig. 2D) expression between groups. In addition, the irreversible suppression of mt CPX4-2 by HFD might further imply another pathway parallel to coenzyme Q_10_ in regulating the expression of different mt CPXs.

### Coenzyme Q_***10***_ treatment reversed mitochondrial biogenesis in aortic perivascular adipose tissue suppressed by high fructose diet ingestion

**Fig. 3:**
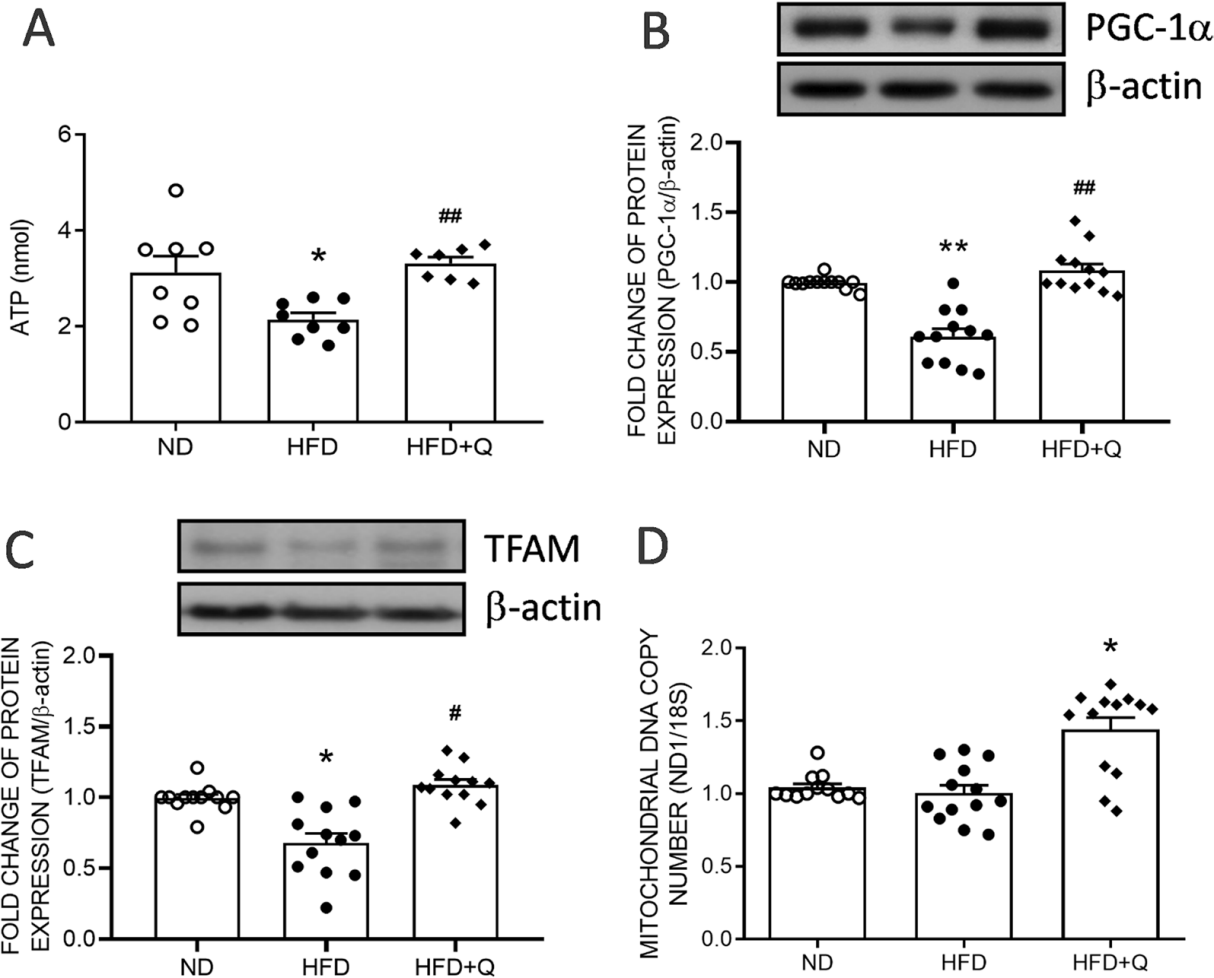
Coenzyme Q_10_ reversed the HFD-downregulated mitochondrial biogenesis regulators in aortic PVAT. **A** The ATP content, representative gel and densitometric analyses of **B** PGC-1α and **C** TFAM as well as **D** mtDNA copy number in the aortic PVAT of the ND, HFD, and HFD + Q groups at the age of 20 weeks. Values are the mean ± SEM, n = 14 for each group. *P < 0.05, **P < 0.01 versus the ND group, and ^***#***^*P* < 0.05 versus the HFD group in the post hoc Tukey’s multiple range test. ND: normal diet, HFD: high fructose diet, HFD + Q: HFD with coenzyme Q_10_. β-actin was used as the internal control for Western blot analyses. PGC-1α: peroxisome proliferator-activated receptor γ coactivator 1 α, TFAM: mitochondrial transcription factor A, mtDNA: mitochondrial DNA, 18S ribosomal RNA (18S) as the internal control of QPCR

Further, the ATP content of PVAT was examined. The result indicated that ATP content of PVAT in the HFD group was significantly reduced when compared with the ND group while coenzyme Q_10_ treatment effectively reversed the ATP level (Fig. 3A). The reduction in mitochondrial mass might be a result of reduced mitochondrial biogenesis. Mitochondrial biogenesis is regulated by PGC-1α. The Western blot results indicated that the expression of PGC-1α was significantly suppressed in the HFD group (Fig. 3B). PGC-1α promotes TFAM expression to maintain mitochondrial biogenesis. Concurrent with the downregulated PGC-1α, the expression of TFAM was significantly decreased in the HFD group (Fig. 3C). On the other hand, the mtDNA copy number was not disturbed by HFD (Fig. 3D). Coenzyme Q_10_ treatment effectively increased the levels of PGC-1α and TFAM and mtDNA copy number (Fig. 3).

### Coenzyme Q_10_ treatment reversed mitochondrial fusion in aortic perivascular adipose tissue suppressed by high fructose diet ingestion

**Fig. 4:**
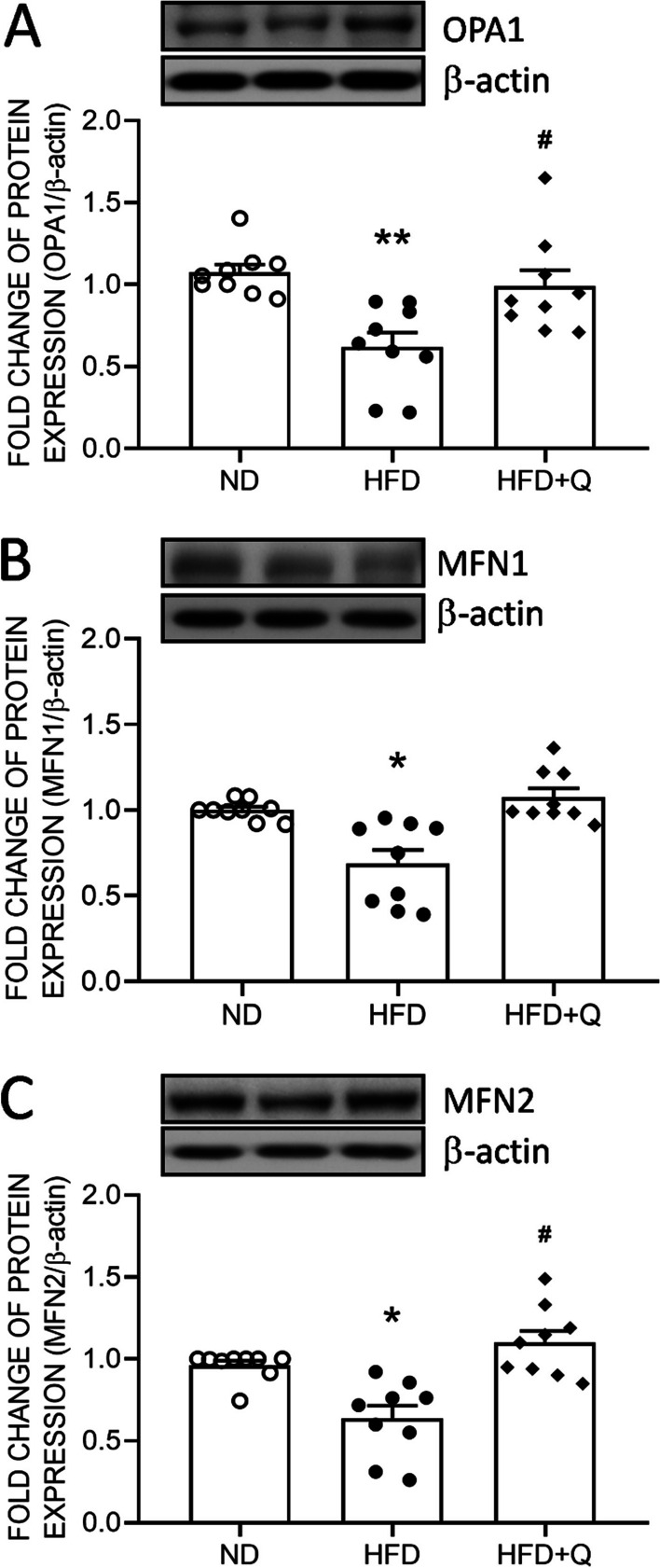
Coenzyme Q_10_ reversed the HFD-induced suppression of mitochondrial fusion in aortic PVAT. Representative gel and densitometric analyses of **A** OPA1, **B** MFN1, and **C** MFN2 in the aortic PVAT of the ND, HFD and HFD + Q groups at the age of 20 weeks. Values are the mean ± SEM, n = 9 for each group. *P < 0.05, **P < 0.01 versus the ND group and ^***#***^*P* < 0.05 versus the HFD group in the post hoc Tukey’s multiple range test. ND, normal diet, HFD, high fructose diet, HFD + Q, HFD with coenzyme Q_10_. OPA1, mitochondrial Dynamin-Like GTPase, MFN, mitofusin, β-actin as the internal control

Mitochondrial dynamics are highly related to mitochondrial mass. The results from Western blot analyses further indicated that the expression of OPA1 (Fig. 4A), MFN1 (Fig. 4B) and MFN2 (Fig. 4C) was downregulated in the PVAT of the HFD group. These results suggested a reduction in mitochondrial fusion by HFD intake. Coenzyme Q_10_ treatment effectively reversed the suppressed OPA1 and MFN2 to the ND level, while an increasing trend of MFN1 was detected in the HFD + Q group (Fig. 4).

### Coenzyme Q_***10***_ treatment reversed phospho-DRP1 expression in aortic perivascular adipose tissue enhanced by high fructose diet ingestion

**Fig. 5:**
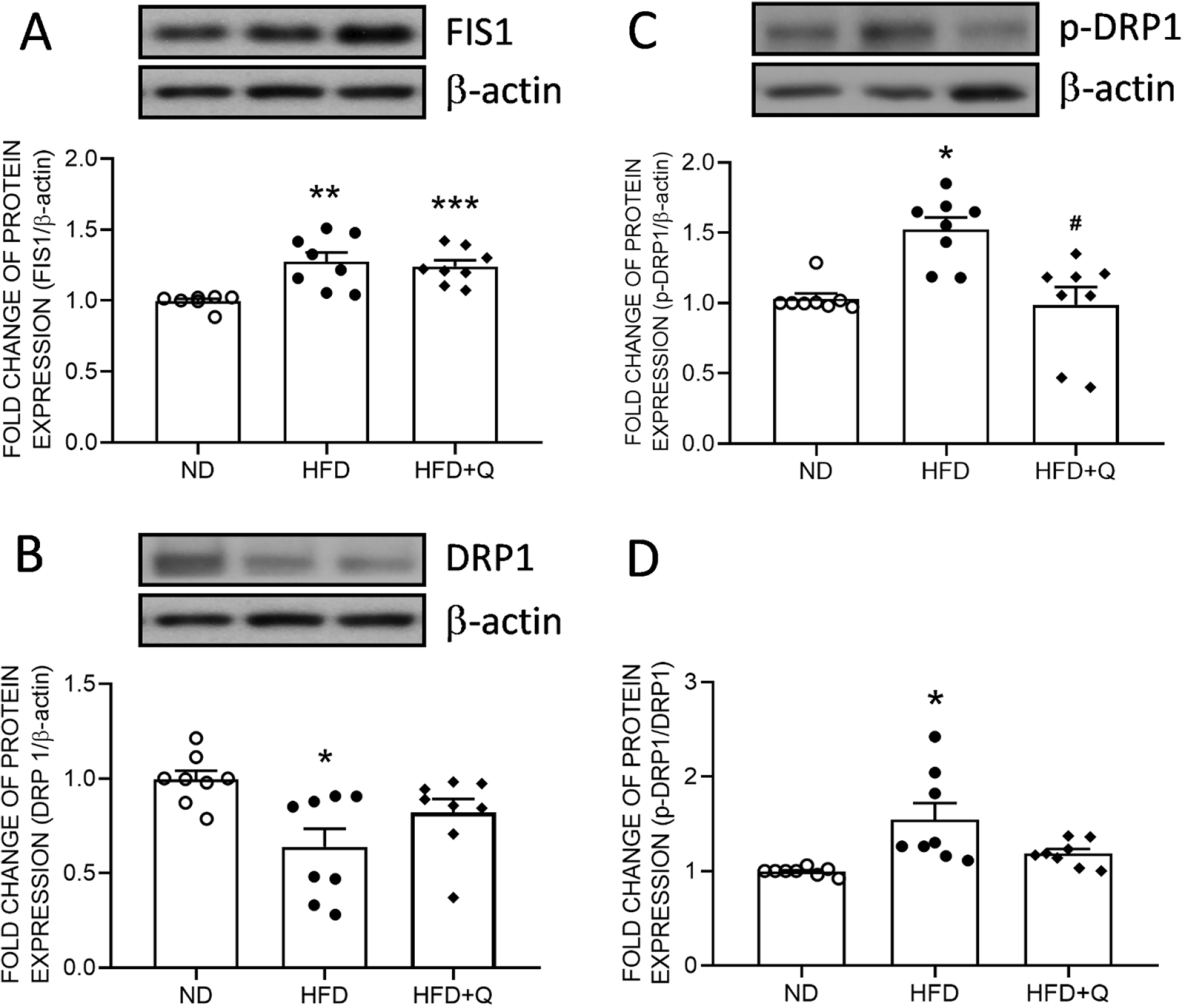
Coenzyme Q_10_ reversed HFD-induced mitochondrial fission in aortic PVAT. Representative gel and densitometric analyses of **A** FIS, **B** DRP1, **C** p-DRP1 and **D** the ratio of p-DRP1/DPR1 in the aortic PVAT of the ND, HFD and HFD + Q groups at the age of 20 weeks. Values are the mean ± SEM, n = 8 for each group. *P < 0.05, **P < 0.01, ***P < 0.001 versus the ND group and ^***#***^*P* < 0.05 versus the HFD group in the post hoc Tukey’s multiple range test. ND, normal diet, HFD, high fructose diet, HFD + Q, HFD with coenzyme Q_10_. FIS1, mitochondrial fission 1 protein, DRP1, dynamin-related protein 1, p-DRP1, phospho-DRP1, β-actin as the internal control

Mitochondrial fusion can be negatively regulated by FIS1 and DRP1 [34]. Western blot analyses further indicated that the expression of FIS1 (Fig. 5A) and phospho-DRP1 (p-DRP1, Fig. 5C) and the ratio of p-DRP1/DRP1 showed a significant increase in the HFD group (Fig. 5D) and were significantly upregulated in the HFD group. On the other hand, the expression of DRP1 was downregulated in the HFD group (Fig. 5B). Coenzyme Q_10_ treatment effectively reversed the suppressed p-DRP1 to the ND level (Fig. 5C). However, coenzyme Q_10_ treatment did not reverse the levels of FIS1, the p-DRP1/DRP1 ratio or total DRP1.

### Coenzyme Q_***10***_ treatment reversed the expression of fibrotic and calcification proteins in aortic perivascular adipose tissue increased by high fructose diet ingestion

**Fig. 6:**
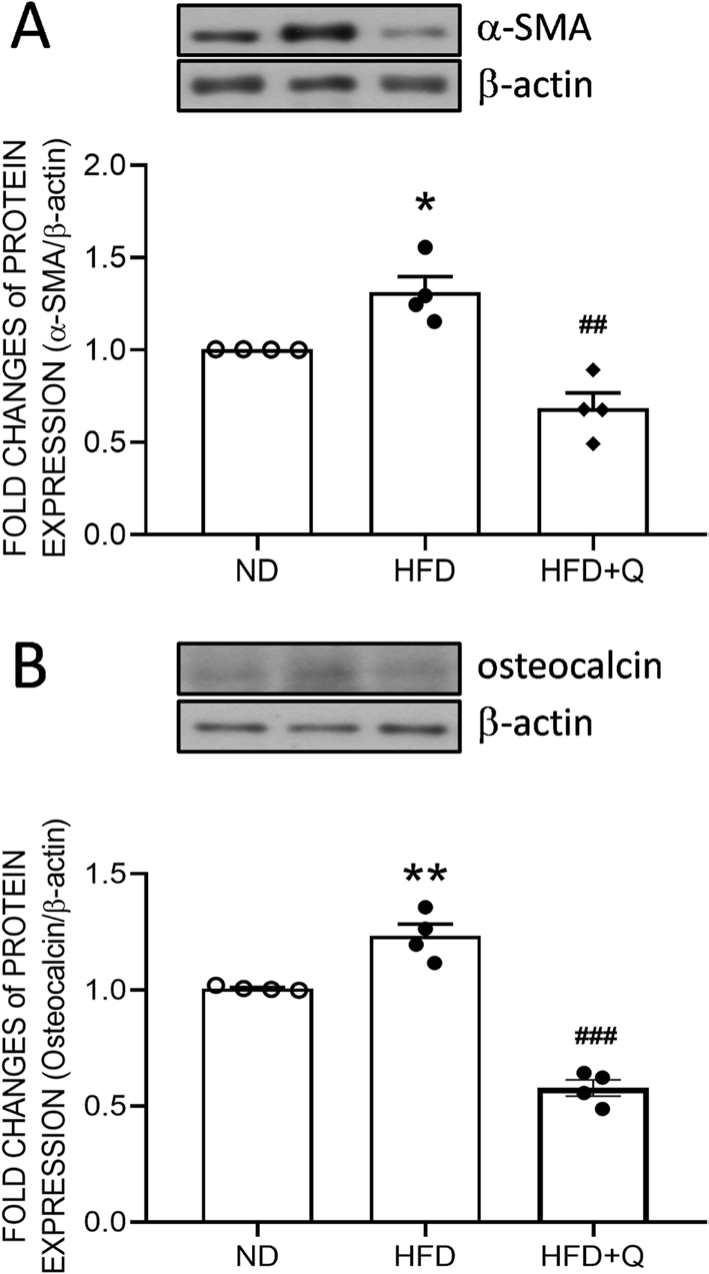
Coenzyme Q_10_ treatment reversed the HFD-induced increase in fibrotic and calcification proteins in aortic PVAT. Representative gel and densitometric analyses of **A** α-SMA and **B** osteocalcin in the aortic PVAT of the ND, HFD and HFD + Q groups at the age of 20 weeks. Values are the mean ± SEM, n = 4 for each group. *P < 0.05, **P < 0.01 versus the ND group and ^##^*P* < 0.01, ^###^*P* < 0.001 versus the HFD group in the post hoc Tukey’s multiple range test. ND, normal diet, HFD, high fructose diet, HFD + Q, HFD with coenzyme Q_10_. α-SMA, α-smooth muscle actin, β-actin as the internal control

Mitochondrial dysfunction of PVAT might promote vascular stiffness [35, 36]. A common marker of fibrosis, α-smooth muscle actin (α-SMA), and an index of calcification, osteocalcin, which is secreted after generation, of aortic PVAT were detected by Western blot analyses. The results indicated that the expression of α-SMA (Fig. 6A) and osteocalcin (Fig. 6B) in aortic PVAT were upregulated in the HFD group. Overgrowth of adipose tissue frequently coincides with cardiovascular disease, implying crosstalk between PVAT and the aorta. The shuttling of specific proteins between PVAT and the aorta might alter aortic function. These results implied that continuous HFD intake may enhance the generation of these stenosis factors in aortic PVAT. Furthermore, the HFD-enhanced α-SMA and osteocalcin were effectively reversed by coenzyme Q_10_ treatment (Fig. 6). These data further suggest the involvement of mitochondrial dysfunction in the characteristic change in PVAT under HFD intake.

### Coenzyme Q_***10***_ treatment reversed the vascular reactivity of the aorta reduced by high fructose diet ingestion

**Fig. 7:**
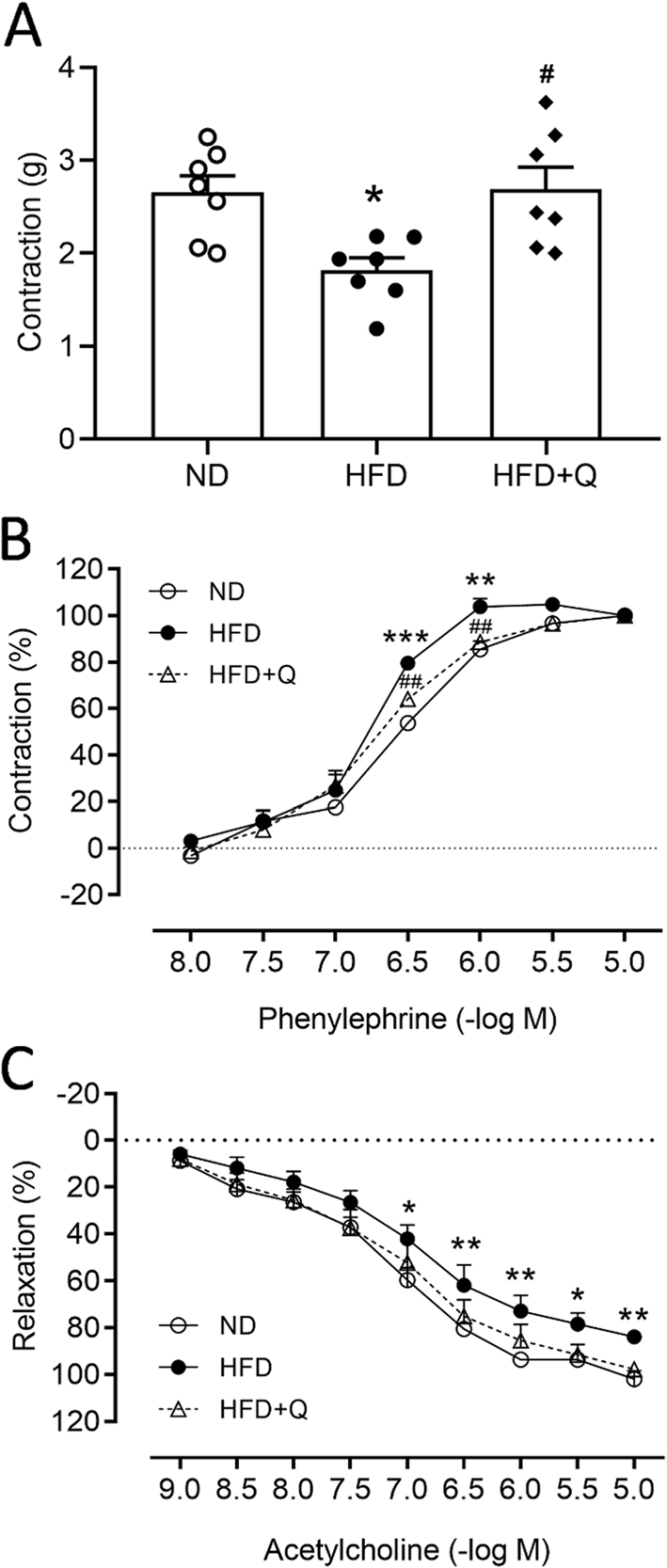
Coenzyme Q_10_ treatment reversed the HFD-impaired vascular reactivity of the thoracic aorta. Vascular responses to **A** KCl, **B** PE, or **C** Ach of the aorta in the ND, HFD and HFD + Q groups at the age of 20 weeks. Values are the mean ± SEM, n = 7 for each group. *P < 0.05, **P < 0.01, ***P < 0.001 versus the ND group and ^***#***^*P* < 0.05, ^***##***^*P* < 0.01 versus the HFD group in the post hoc Tukey’s multiple range test. ND, normal diet, HFD, high fructose diet, HFD + Q, HFD with coenzyme Q_10_. PE, phenylephrine, Ach, acetylcholine

A decrease in vascular reactivity is an index of the initiation of aortic stenosis. PVAT regulates vascular functions [13, 37–39]. The results from the aortic reactivity further indicated that the sensitivity of vascular contraction to KCl was decreased in the HFD group in comparison to the ND group (Fig. 7A). Contractions to phenylephrine (PE) were significantly enhanced in the aortic rings isolated from the HFD group (Fig. 7B; at − log_10_ 6.5 M). These data indicated that the sensitivity of receptor-independent vasocontraction was reduced and the sensitivity of adrenergic receptor-dependent vasocontraction was enhanced as early as 3 months after HFD intake. On the other hand, endothelium-dependent relaxations to acetylcholine (Ach) were significantly impaired in the HDF group, and there was a significant reduction in maximal relaxation in comparison with the ND group (Fig. 7C). Coenzyme Q_10_ treatment effectively reversed HFD-impaired vascular contraction and relaxation.

## Discussion

In this study, we provide novel evidence to suggest that overconsumption of fructose for 3 months contributed to aortic PVAT whitening, which impaired aortic reactivity in adult male rats. In the PVAT, the downregulation of mitochondrial biogenesis regulators was concurrent with reduced UCP1 and mitochondrial mass. Moreover, an imbalance of mitochondrial fusion and fission, including decreased fusion and enhanced fission, was detected in the whitened PVAT. Concurrently, the expression of stenosis factors was significantly enhanced by HFD. Coenzyme Q_10_ treatment effectively reversed the indices of brown adipose tissue, mitochondrial biogenesis, mitochondrial fusion and fission. Most importantly, the stenosis factors were reduced, and the aortic reactivity was effectively reversed. These results unveiled the importance of mitochondrial dynamics in resisting adipose whitening. Furthermore, these results revealed the importance of PVAT in aortic reactivity in addition to bloodborne factors.

Excessive fructose intake has been known to induce the progression of metabolic disorders, including obesity [40], metabolic syndrome [41] and cardiovascular diseases [42–44]. Consistently, our study demonstrated that HFD intake for 3 months was capable of inducing hyperglycemia, hyperinsulinemia and dyslipidemia. Intriguingly, HFD ingestion for 3 months did not induce central obesity. HFD consumption might predispose to disturb metabolic status, for instance, a shift of aortic PVAT from BAT to WAT in 3 months. According to the gross external phenotype (shown below), there were no significant differences between groups. At the cellular molecular level, the UCP1 level and mitochondrial mass are significantly reduced by HFD intake. It is reasonable that the changes at the molecular level occur earlier than the gross phenotypes. Therefore, we consider that the induced metabolic changes after 3 months of HFD intake might be at the early stage.

Aortic PVAT is phenotypically and functionally close to brown adipose tissue [9, 10] containing ample UCP1 and densely packed mitochondria. When adipocytes convert from a brown to white phenotype, the cell contents of UCP1 and the mitochondrial mass are decreased [45]. In this study, we found that HFD intake for 3 months resulted in UCP1 downregulation of aortic PVAT. UCP1 is located in the mitochondrion. The decrease in UCP1 implies a reduced mitochondrial mass. The immunofluorescence images of MitoTracker staining further indicated that mitochondrial mass was reduced in the aortic PVAT of the HFD group. In combination with UCP1 downregulation, these results revealed that HFD intake induced aortic PVAT whitening in 3 months. PVAT whitening can be induced by multiple factors, such as leptin resistance, β-adrenergic signaling impairment, and lipase deficiency. Each of these factors is related to mitochondrial dysfunction [46–49]. The downregulation of the mitochondrial respiratory complexes in the aortic PVAT further uncovered a suppression of mitochondrial mass and the deficiency of the respiratory machinery under HFD intake.

Mitochondrial biogenesis actively regulates mitochondrial mass. The PGC-1α and TFAM downregulation further linked the suppression of mitochondrial biogenesis to PVAT whitening. mtDNA copy number is considered one index of mitochondrial biogenesis. Inconsistent to the downregulations of PGC-1α and TFAM as well as respiratory complexes, the mtDNA copy number was not significantly disturbed by HFD. The mtDNA copy number is positively related to lipogenesis [50]. These lines of evidence imply that HFD consumption might be prone to reduce mitochondrial mass instead of inducing lipogenesis in 3 months.

However, the downregulation of MFN2 has been demonstrated to contribute to adipose tissue whitening [21]. Consistent with this study, our data indicated that the expression of MFN2 was suppressed by HFD intake. Moreover, we found that the other fusion factors, OPA1 and MFN1, were suppressed in the HFD group. These results further suggested that the machinery of mitochondrial fusion was reduced by HFD intake. The MFN2 and coenzyme Q_10_ interaction has been reported to maintain cellular respiration [51]. Similarly, treatment with coenzyme Q_10_ effectively reversed the HFD-induced reduction in the expression of these mitochondrial fusion factors. These lines of evidence further suggest that mitochondrial fusion protects against the whitening of PVAT. Nonetheless, the underlying mechanism(s) of HFD-associated downregulation of these fusion proteins await further investigation.

Instead of promoting mitochondrial biogenesis, DRP1-FIS1 binding at the periphery of mitochondria facilitates the division of damaged mitochondria [22]. Recently, an increase in Drp1 protein and decreases in MFN2 and OPA1 protein expression were observed in the white adipose tissue of *ob*/*ob* mice [52], implying the involvement of mitochondrial fission in the progression of adipose whitening. Consistent with these studies, our data indicated that DRP1 activation and FIS1 expression were enhanced after 3 months of HFD intake. These lines of evidence imply that HFD-enhanced mitochondrial fission might negatively regulate mitochondrial biogenesis. In addition, coenzyme Q_10_ showed no effect on reversing the induced FIS1 expression and the ratio of p-DRP1/DRP1. These results implied that the HFD-increased mitochondrial fission might be upstream of or parallel to coenzyme Q_10_ signaling. Of course, it is also possible that the decrease in fusion and increase in fission are merely the results of overnutrition, while sustained dysregulation of mitochondrial dynamics compromises mitochondrial biogenesis.

The paracrine effects of PVAT on vasoreactivity have been well documented in human and rodent models of high-fat diet [53]. PVAT possesses both metabolic and endocrine functions in regulating vascular functions, including vasorelaxation [54] and vasodilation [55–57] under physiological conditions. The underlying mechanisms include transferable adipocyte-derived relaxing factor (ADRF) to induce endothelium-dependent vasorelaxation while PVAT-derived reactive oxygen species (ROS) directly inhibit SMC contraction by activating soluble guanylyl cyclase [58]. The effect of a high-fructose diet on endothelium-independent relaxation has been documented by Xue et al. [59]. Accordingly, endothelium-independent relaxation was not significantly altered by HFD. Based on the spatial relations, PVAT is closer to smooth muscle than the endothelial layer. The whitened PVAT-derived α-SMA and osteocalcin in this study might initiate an adverse effect on smooth muscle earlier rather than on endothelium. The temporal progression is currently speculative and requires further study. In this study, we further emphasize that the adverse effects of HFD at an early stage might initiate a pro-stenosis microenvironment around the aorta through the whitening of PVAT.

Under high fructose intake, we found a higher response to PE-induced vasoconstriction and a lower sensitivity to Ach-induced vasorelaxation concurrent with the upregulation of α-SMA and osteocalcin in PVAT. In particular, osteocalcin is an extracellular protein involved in calcification. These results are similar to a previous human cohort study reporting that thoracic peri-aortic fat is associated with aortic calcification [60]. These lines of evidence implied that continuous HFD intake might alter the metabolic status of PVAT to alter the secretion of stenosis factors, such as osteocalcin, leading to reduced vascular reactivity of the aorta in 3 months. It is conceivable that a long-term accumulation of paracrine stress on vascular reactivity might eventually lead to aortic vascular disease. On the other hand, the involvement of elevating inflammation, and dysregulation of sympathetic activity in BAT whitening has been well-documented [45, 61] and these factors contribute to calcification [62, 63]. Whether the pro-stenosis factor is primary to or parallel to these pathological progressions remain unclear. Moreover, the involvement of the blood-born effect on the aorta cannot be ruled out owing to the limitation of the study design.

The dosage of CoQ10 is based on the safety range of clinical instruction. We applied this suggested dosage to verify the effect of the CoQ10 supplement on preventing PVAT whitening. The positive results suggest that 10 mg/100 mL for one month should be able to reverse the HFD-induced adipose whitening in aortic PVAT. Coenzyme Q10 is an electron transporter of the mitochondrial electron transport chain. Thus, CoQ10 was applied to maintain mitochondrial function against PVAT whitening in this study. In addition, CoQ10 acts as a natural antioxidant to neutralize free radical species that activate NF-κB resulting in reducing the levels of cytokines, such as TNF-α and IL-6 [64, 65]. The study design didn’t route out the beneficial effects of CoQ10 detected in this study on the anti-inflammation. This current speculation requires further study.

Together, our study indicated that HFD intake for 3 months could induce the whitening of aortic PVAT, which might result in aortic stenosis. Coenzyme Q_10_ can reverse HFD-induced thoracic aortic PVAT whitening and reduce aortic reactivity by maintaining mitochondrial dynamics.

## Abbreviations

α-SMA: α-Smooth muscle actin
BAT: Brown adipose tissue
DAPI: 4′,6-Diamidino-2-phenylindole
DRP1: Dynamin-related protein 1
HFD: High fructose diet
FBG: Fasting blood glucose
FBI: Fasting blood insulin
FBTG: Fasting blood triglyceride
ELISA: Enzyme-linked immunosorbent assay
FIS1: Mitochondrial fission 1 protein
IACUC: Institutional animal care and use committee
MetS: Metabolic syndrome
MFN: Mitofusin
mt CPX: Mitochondrial respiratory complex
mtDNA: Mitochondrial DNA
ND: Regular diet
OPA1: Optic atrophy 1
OCT: Optimal cutting temperature
PGC-1α: Peroxisome proliferator-activated receptor γ coactivator 1α
PVAT: Perivascular adipose tissue
PVDF: Polyvinylidene difluoride
Q: Coenzyme Q_10_
ROS: Reactive oxygen species
SD: Sprague-Dawley
TG: Triglycerides
TFAM: Mitochondrial transcription factor A
UCP: Uncoupling protein
WAT: White adipose tissue
